# The Relation between Sarcopenia and Mortality in Patients at Intensive Care Unit

**DOI:** 10.1155/2018/5263208

**Published:** 2018-02-12

**Authors:** Mehmet Toptas, Mazhar Yalcin, İbrahim Akkoc, Eren Demir, Cagatay Metin, Yildiray Savas, Muhsin Kalyoncuoglu, Mehmet Mustafa Can

**Affiliations:** ^1^Department of Anesthesiology, Haseki Training and Research Hospital, Istanbul, Turkey; ^2^Department of Radiology, Haseki Training and Research Hospital, Istanbul, Turkey; ^3^Department of Cardiology, Haseki Training and Research Hospital, Istanbul, Turkey

## Abstract

**Background and Aim:**

Psoas muscle area (PMA) can reflect the status of skeletal muscle in the whole body. It has been also reported that decreased PMA was associated with postoperative mortality or morbidity after several surgical procedures. In this study, we aimed to investigate the relation between PMA and mortality in all age groups in intensive care unit (UNIT).

**Materials and Method:**

The study consists of 362 consecutive patients. The demographic characteristics of patients, indications for ICU hospitalization, laboratory parameters, and clinical parameters consist of mortality and length of stay, and surgery history was obtained from intensive care archive records.

**Results:**

The mean age was 61.2 ± 18.2 years, and the percentage of female was 33.3%. The mean duration of stay was 10.3 ± 24.4 days. Exitus ratio, partial healing, and healing were 25%, 70%, and 5%, respectively. The mean right, left, and total PMA were 8.7 ± 3.6, 8.9 ± 3.4, and 17.6 ± 6.9, respectively. The left and total PMA averages of the nonoperation patients were statistically significantly lower (*p* = 0.021  *p* = 0.043). The mean PMA between the ex and recovered patients were statistically significantly lower (*p* = 0.001, *p* = 0.001, *p* < 0.001). Dyspnoea, renal insufficiency, COPD, transfusion rate, operation rate, ventilator needy, and mean duration of hospitalization were statistically significant higher in patients with exitus. There is a significant difference in operation types, anesthesia type, and clinic rates.

**Conclusion:**

Our data suggest that sarcopenia can be used to risk stratification in ICU patients. Future studies may use this technique to individualize postoperative interventions that may reduce the risk for an adverse discharge disposition related to critical illness, such as early mobilization, optimized nutritional support, and reduction of sedation and opioid dose.

## 1. Introduction

Every year, millions of patients are followed up in the intensive care unit (ICU) in postoperative period or various diseases and some of these patients died. There are many parameters used to determine mortality in patients in the ICU: age, gender, chronic illness, acute physiological values (vital findings), and laboratory values such as serum creatinine level, troponin, lactate, and serum cystatin C [1]. None of these parameters directly correlated with mortality. Therefore, the parameters that can predict mortality are being investigated [2].

Sarcopenia, which means decreasing volume and function of muscle tissue as it ages, generally refers to the reduction of the physiological reserve in the body [3]. Previous studies have shown that sarcopenia is associated with chronic heart failure, postoperative status, after surgery, trauma, extended mechanical ventilation, longer hospital stays, and mortality [4–8].

Psoas muscle area (PMA) because it is a core muscle can reflect the status of skeletal muscle in the whole body [5, 8]. It has been also reported that decreased PMA, as a marker of sarcopenia, was associated with postoperative mortality or morbidity after several surgical procedures [5]. While there were relatively more data available about the prognostic value of sarcopenia in patients suffered surgery, trauma, or cancer, its importance for patients with mortality in whole ICU patients, not only the elderly, was little [5, 6]. So, in this study, we aimed to investigate the relation between PMA and mortality in all age groups with intensive care unit.

## 2. Methods

Our study has cross-sectional design and included patients in intensive care unit of our hospital between May 2012 and May 2017. The relationship between the incidence of in-hospital mortality and sarcopenia level was investigated. Three hundred sixty-two ICU patients were included in the study. CT scan images were used to determine the quantity of skeletal muscle. The skeletal muscle cross-sectional area (cm2) was manually measured at the caudal end of the third lumbar vertebra. Computed tomography images were used to determine the quantity of skeletal muscle. CT scans were retrieved to measure right and left psoas muscle area, to obtain the total psoas area. The PSA was measured by an observer who was blinded to the outcome and disease severity.

For each patient record, following data were collected including age, sex, smoking, number of comorbidities presenting, ASA score, and Glasgow Coma Scale Score. During the length of stay in ICU all laboratory measurements, the reasons of admission to ICU (urgent, surgical, or internal reasons), type of anesthesia, ventilator requirement in ICU, transfusion requirement, duration of stay, and final status were recorded. The relationship between each of these parameters and the psoas muscle area was evaluated separately.

Statistical analyses were performed by SPSS 15.0 for Windows. In addition to descriptive statistics, mean, standard deviation, and minimum and maximum are used for numeric variables, and number and percentage for categorical variables. The Kolmogorov-Smirnov test was used to assess whether the variables were normally distributed. Student's *t*-test or Mann–Whitney *U* test was used to compare the continuous variables between the groups according to whether it was normally distributed or not. Comparisons of ratios in groups were made with Chi Square Analysis. Binary logistic regression analysis (backward stepwise method) was performed to identify independently associated factors with mortality. Variables with a *p* value < 0.25 in univariate analysis were incorporated in the binary logistic regression analysis. Statistical significance level of alpha was accepted as *p* < 0,05.

## 3. Results

The general and operative characteristics of the study group are summarized in Table 1. The mean age was 61.2 ± 18.2 years, and the percentage of men was 33.3%. The mean duration of stay was 10.3 ± 24.4 days. Exitus ratio, partial healing, and healing were 25%, 71%, and 4%, respectively. The laboratory and PMA evaluations of the study group are summarized in Tables 2 and 3, respectively. The mean right, left, and total PMA were 8.7 ± 3.6, 8.9 ± 3.4, and 17.6 ± 6.9, respectively.

The mean right, left, and total PMA in patients with dyspnoea, COPD, CHF, female gender, and nonsmokers were statistically significantly lower (Table 4). The left and total PSOAS muscle area averages of the nonoperation patients were statistically significantly lower (*p* = 0.021  *p* = 0.043) (Table 5). We have obtained statistically significant difference in left PMA averages in anesthesia groups (*p* = 0.045) (Table 5). Patients' left PMA averages, who had regional anesthesia, are lower than the others who has not taken anesthesia (*p* = 0.017) (Table 5). The mean PMA between the ex and recovered patients was statistically significantly lower (*p* = 0.001  *p* = 0.001  *p* < 0.001) (Table 6).

There was a statistically significant positive correlation with glucose, ALT, CK, WBC, RBC, Hg, Hct and negative correlation with age, ASA Score, urea, ALP, CRP, and right, left, and total PSOAS muscle area (Table 7). Dyspnoea, renal insufficiency, COPD, transfusion rate, operation rate, ventilator needy, and mean duration of hospitalization were statistically significant higher in patients with exitus (Table 8). There is a significant difference in operation types, anesthesia type, and clinic rates (Table 9). In the multiple logistic regression analysis, COPD (OR: 0.307 (CI: 0.113–0.835), *p* = 0.021) and total PMA (OR: 0.812 (CI: 0.741–0.890), *p* < 0.000) were found to be independently associated with mortality (Table 10).

## 4. Discussion

Fragility is explicitly undefined and is known as failure to maintain homeostasis due to insufficient response to some stressors associated with reduced reserve in the multiple organ system [5–13]. It has been reported that frailty was predictor of mortality and morbidity than chronological age [14–17]. Some parameters such as physical activity level, unintentional weight loss, slow walking speed, fatigue, loss of physical strength, comorbid medical conditions, loss of independence for activities of daily living, low albumin levels, and cognitive impairments have been described in evaluating and defining the fragility [13, 17–20]. Present study demonstrated that decreased skeletal muscle mass was a significant predictor of in-hospital mortality in the sample of patients admitted to a tertiary medical center ICU. Sarcopenia, a frailty risk factor of particular interest is age-related loss of muscle mass and/or strength and performance and has been closely related to reduced quality of life, geriatric syndromes, greater morbidity, and mortality [20–24]. PMA because it is a core muscle can reflect the status of skeletal muscle in the whole body [12]. It has been reported that decreased PMA, as a marker of sarcopenia, was associated with postoperative mortality or morbidity after abdominal aortic aneurysm repair, liver transplantation, pancreatic cancer resection, adrenocortical cancer resection, colorectal cancer resection, radical cystectomy, and surgical or percutaneous aortic valve replacement [6]. It was also reported that direct measurement of muscle mass can give the best information about the physiological reserve. In some studies, measurement of the psoas muscle at the level of the third lumbar vertebra (L3) with CT has been used to determine the physiological reserve prior to some operations, such as liver transplantation [5, 12]. The prognostic value of sarcopenia has been determined for patients after surgery and trauma or with cancer. Moreover, Mueller et al. found that sarcopenia assessed with ultrasound predicts adverse discharge disposition as well as duration of hospitalization [9]. However their study did not conclude any comments on hospital mortality associated with sarcopenia. This is the first study, to the best of our knowledge, to examine the implications of sarcopenia evaluated by cross-sectional PMA, on mortality in ICU patients.

Single-slice muscle area has been found to be associated with total body muscle mass and as a predictor for the postoperative outcomes after various surgical procedures [5]. Similar to our study, Weijs et al. demonstrated a relationship between the low skeletal muscle mass assessed by CT and extended mechanical ventilation, longer hospital stays, and mortality [7]. In line with our findings, Moisey et al. recently found that low muscle mass as assessed by CT scans was associated with mortality in 149 injured elderly ICU patients [8]. Besides that, in contrast to Moisey et al.'s population, our findings are made in an ICU representative age group, and not in an elderly and only traumatic population. It is difficult to make these measurements in order to estimate skeletal muscle mass. It is often required as part of the initial study in patients. Therefore, it is possible that, in this patient population, an early evaluation of muscle mass and muscle cross-sectional view provide an objective method that estimates lean muscle easily obtained. So, in this study, CT was used to estimate total muscle mass determined by cross-sectional area of the psoas muscle as a marker of sarcopenia.

Risk estimation, prediction, and the results achieved by bringing the perspective of resource allocation and assessment of quality of health services are important [25]. The individual approach to patient care in the intensive care unit following the treatment plan for each patient to identify the optimal risk-benefit ratio should be evaluated. Clinical characteristics that impact the mortality rate and the length of the hospital stay include multiple comorbidities such respiratory, cardiac, renal, and infectious problems. Several factors such as comorbidity can be evaluated in a variety of ways that help predict prognosis [26–32]. In spite of the apparent variability between observers, ASA classification has been widely accepted in the prediction of morbidity and mortality [28–33].

Regardless of anesthesia application, it is expected to increase mortality and morbidity in patients with systematic disease. [32, 34]. Therefore, patients in bad health condition are expected to have higher rates of admission to the ICU [32, 35–37]. Our study showed that mortality, length of stay in the ICU, and duration of mechanical ventilation increased as PMA decreased.

All these scoring systems can help in the prediction of patient program, although it should be noted that the prognosis for each patient may be different [38]. Frail patients may have a lower functional capacity and decreased ability to mobilize at baseline. Thus, they are vulnerable against severe physiologic stressors, predisposing them to functional dependence at discharge and death.

In the current study, we confirmed associations between decreasing muscle mass and increased mortality in ICU patients. According to the results of our study, there was a close relationship between PMA values and mortality in ICU patients independent of other variables. Thus, fragility was quantitatively calculated and the prevalence of ICU patients emerged.

Because skeletal muscle atrophy can cause physical decline such as impaired cytokine [39, 40] and insulin signaling [41–43] that may result in glucose intolerance, we speculate that stratification by muscle mass may reflect physical condition. Due to the design of the study, sarcopenia and the relationship between the mechanisms of poor prognosis cannot be determined with certainty. However, the results of the current study emphasize mass and function of skeletal muscle in ICU patients.

As a result, it is appropriate to consider that frailty may be important in the treatment options and follow-up of the patients. While PMA is quantitatively indicative of frailty, CT exposure to PE patients is already present, but exposure to radiation and contrast remains. For this reason, it is plausible to plan studies to understand whether clinical frailty scores, such as Fried scoring or simple “FRAIL” Questionnaire Screening, on the outcome of PMA can influence the prognosis of ICU patients and lead to treatment [16, 18]. Mortality during hospital stay or functional information about the risk of dependence makes informed decisions about the goals of care may help.

The limitations of the present study are the lack of outpatient and surveys. Moreover, this is a retrospective analysis that could not lead to the conclusion which might only represent the background for future perspective studies that will confirm the impact of sarcopenia in ICU. Moreover, due to its retrospective nature, we could not assess our nutritional status of patients. We can clearly show the relationship between sarcopenia and malnutrition status of patients with tests such as the Mini Nutritional Assessment in further studies. Consequently, our data suggest that sarcopenia can be used in risk stratification in ICU patients. CT is a valid and simple technique that could also be used for longitudinal assessment of treatment success. Future studies may use this technique to individualize postoperative interventions that may reduce the risk for an adverse discharge disposition related to critical illness, such as early mobilization, optimized nutritional support, and reduction of sedation and opioid dose.

## Figures and Tables

**Table 1:** The general and operative characteristics of the study group.

*Age *Mean ± SD (Min–Max)	61,2 ± 18,2 (18–92)
*Gender n* (%)	
Male	238 (65,7)
Female	124 (34,3)
*Smoking*	227 (62,7)
*Comorbidity n* (%)	
Diabetes	116 (32,0)
Dyspnea	226 (62,4)
Renal insufficiency	122 (33,7)
Cancer	156 (43,1)
KKY	115 (31,8)
KOAH	68 (18,8)
*Transfusion n* (%)	220 (60,8)
*Operation n* (%)	
Urgent	110 (30,4)
Elective	123 (34,0)
None	129 (35,6)
*Type of anesthesia n* (%)	
General	219 (60,5)
Regional	14 (3,9)
None	129 (35,6)
*Clinic n* (%)	
Urgent	62 (17,1)
Surgical	247 (68,2)
Internal	53 (14,6)
*ASA Score n* (%)	2,5 ± 1,0 (1–5)
1	44 (18,0)
2	75 (30,7)
3	90 (36,9)
4	32 (13,1)
5	3 (1,2)
*GCS *Mean ± SD (Min–Max)	13,3 ± 3,2 (3–15)
0–8	40 (11,1)
>9	319 (88,9)
*Ventilator requirement (day) *Mean ± SD (Min–Max)	14,4 ± 31,7 (1–322)
*Duration of stay *Mean ± SD (Min–Max)	10,3 ± 24,4 (1–307)
*Final status n* (%)	
Ex	91 (25,1)
Partial recovery	256 (70,7)
Recovery	15 (4,1)

**Table 2:** Laboratory parameters of the study population.

	Mean ± SD	Min–Max
Glucose	152,3 ± 69,3	51–721
Urea	65,8 ± 59,3	3,3–432
CRE	1,67 ± 2,23	0,18–23,2
AST	126,3 ± 369,7	4,3–3511
ALT	77,0 ± 218,5	1–2499
GGT	74,0 ± 121,4	5–1000
ALP	106,2 ± 104,8	23,3–902
T. Protein	5,0 ± 1,0	2,9–7
Albumin	2,6 ± 0,6	0,9–5
CK	259,5 ± 498,4	7,8–4838
Sodium	136,4 ± 8,1	25–156
Potassium	4,4 ± 3,0	2,06–59
Calcium	7,7 ± 1,0	2,5–12
CRP	121,2 ± 108,1	0,2–564
WBC	14,7 ± 10,8	1,9–86,1
RBC	3,8 ± 0,8	1,4–6
HG	10,5 ± 2,2	2,3–17,7
HCT	32,3 ± 6,6	10,9–54,2
PLT	230036,1 ± 143536,0	11000–1233000
Neutrophil	12,5 ± 9,6	1–83,5
Lymphocyte	1,3 ± 1,3	0–11,9

CRE: Creatinine, GGT: Gama glutamyl transferase, ALP: Alkanine Phosphatase, CK: Cratinine kinase, AST: Aspartate dehydrogenase ALT: Alanine dehydrogenase, CRP. Cerum reactive proeiin, Wbc: White blood cells, RBC: Red blood cells, HG: Hemoglobin, HCT: Hematocrit, PLT: PlateletLDH: Lactate dehydrogenase.

**Table 3:** Physical characteristics of study population, PSOAS muscle area.

	Mean ± SD	Min–Max
Right PSOAS	8,7 ± 3,6	1,55–28,35
Left PSOAS	8,9 ± 3,4	1,23–22,4
Total PSOAS	17,6 ± 6,9	2,78–45,94

**Table 4:** Association of the demographic parameters and the physical characteristics in study population.

	Right PSOAS	Left PSOAS	Total PSOAS
	Mean ± SD	Mean ± SD	Mean ± SD
*Gender*			
Male	9,88 ± 3,55	10,14 ± 3,30	20,01 ± 6,65
Female	6,33 ± 2,32	6,53 ± 2,19	12,86 ± 4,32
	<0,001	<0,001	<0,001
*Smoking*			
Yes	9,26 ± 3,51	9,57 ± 3,26	18,83 ± 6,59
No	7,65 ± 3,54	7,78 ± 3,40	15,44 ± 6,79
	<0,001	<0,001	<0,001
*Diabetes Mellitus*			
Yes	8,26 ± 2,57	8,54 ± 2,55	16,80 ± 4,97
No	8,85 ± 3,98	9,07 ± 3,75	17,92 ± 7,56
	0,488	0,497	0,473
*Dyspnea*			
Yes	8,02 ± 3,52	8,26 ± 3,31	16,28 ± 6,63
No	9,72 ± 3,49	9,97 ± 3,35	19,69 ± 6,71
	<0,001	<0,001	<0,001
*Renal insufficiency*			
Yes	8,28 ± 3,38	8,54 ± 2,96	16,83 ± 6,06
No	8,86 ± 3,70	9,09 ± 3,63	17,93 ± 7,21
	0,212	0,303	0,259
*Cancer*			
Yes	8,39 ± 3,00	8,68 ± 2,88	17,07 ± 5,78
No	8,87 ± 3,99	9,07 ± 3,78	17,93 ± 7,56
	0,701	0,790	0,710
*CHF*			
Yes	7,98 ± 3,41	8,30 ± 2,92	16,29 ± 6,08
No	8,98 ± 3,65	9,18 ± 3,60	18,16 ± 7,12
	0,005	0,030	0,013
*COPD*			
Yes	7,76 ± 3,68	7,77 ± 2,75	15,53 ± 6,10
No	8,87 ± 3,55	9,16 ± 3,51	18,03 ± 6,94
	0,004	0,004	0,003

CHF: congestive heart failure; COPD: chronic obstructive pulmonary disease.

**Table 5:** Association between the clinical parameters and the physical characteristics in study population.

	Right PSOAS	Left PSOAS	Total PSOAS
	Mean ± SD	Mean ± SD	Mean ± SD
*Operation*			
Yes	8,86 ± 3,57	9,19 ± 3,48	18,04 ± 6,93
No	8,31 ± 3,63	8,39 ± 3,27	16,69 ± 6,66
	0,091	0,021	0,043
*Operation*			
Urgent	8,82 ± 3,64	9,17 ± 3,59	17,99 ± 7,06
Elective	8,90 ± 3,53	9,20 ± 3,39	18,10 ± 6,84
None	8,31 ± 3,63	8,39 ± 3,27	16,69 ± 6,66
	0,228	0,067	0,124
*Type of anesthesia*			
General	8,92 ± 3,63	9,25 ± 3,53	18,17 ± 7,03
Regional	7,95 ± 2,45	8,13 ± 2,44	16,08 ± 4,82
None	8,31 ± 3,63	8,39 ± 3,27	16,69 ± 6,66
	0,188	0,045	0,087
*Clinic*			
Urgent	8,60 ± 3,33	8,93 ± 3,42	17,50 ± 6,63
Surgical	8,72 ± 3,55	9,06 ± 3,47	17,78 ± 6,91
Internal	8,45 ± 4,13	8,16 ± 3,14	16,63 ± 6,88
	0,644	0,181	0,402
*Transfusion*			
Yes	8,65 ± 3,94	8,87 ± 3,61	17,52 ± 7,35
No	8,68 ± 3,02	8,96 ± 3,12	17,63 ± 6,03
	0,404	0,518	0,442
*GCS*			
0–8	8,62 ± 4,48	8,13 ± 3,13	16,71 ± 7,20
>9	8,65 ± 3,46	8,99 ± 3,46	17,64 ± 6,80
	0,562	0,116	0,256
*Final status*			
Recovery	8,95 ± 3,48	9,22 ± 3,44	18,17 ± 6,79
Ex	7,80 ± 3,83	7,96 ± 3,21	15,75 ± 6,77
	0,010	0,009	0,006
*Final status*			
Ex	7,80 ± 3,83	7,96 ± 3,21	15,75 ± 6,77
Partial recovery	8,87 ± 3,43	9,13 ± 3,38	18,00 ± 6,67
Recovery	10,40 ± 4,10	10,73 ± 4,20	21,13 ± 8,19
	0,001	0,001	<0,001

GCS: Glasgow Coma Scale.

**Table 6:** Association between the PMA and mortality in study population.

	Recovery	Exitus	*p*
	Median ± SD (median)	Ave. ± SD (median)	
Right PSOAS	8,95 ± 3,48 (9)	7,79 ± 3,83 (7)	0,001
Left PSOAS	9,22 ± 3,44 (9)	7,95 ± 3,21 (7)	0,001
Total PSOAS	18,2 ± 6,8 (18)	15,7 ± 6,8 (14)	0,001

**Table 7:** 

	Right PSOAS		Left PSOAS		Total PSOAS	
	rho	*p*	rho	*p*	rho	*p*
Age	−0,365	<0,001	−0,359	<0,001	−0,370	<0,001
ASA Score	−0,209	0,001	−0,182	0,004	−0,200	0,002
GCS	0,090	0,088	0,119	0,024	0,110	0,037
Ventilator requirement (day)	−0,157	0,033	−0,140	0,057	−0,149	0,043
Duration of stay mean ± SD	−0,089	0,091	−0,075	0,155	−0,084	0,109
Glucose	0,165	0,002	0,139	0,008	0,158	0,003
Urea	−0,124	0,018	−0,118	0,026	−0,119	0,024
CRE	0,044	0,411	0,023	0,676	0,038	0,487
AST	0,045	0,415	0,006	0,910	0,028	0,616
ALT	0,197	<0,001	0,165	0,003	0,189	0,001
GGT	−0,036	0,549	−0,049	0,423	−0,040	0,514
ALP	−0,180	0,004	−0,191	0,002	−0,189	0,003
T. protein	−0,050	0,579	−0,068	0,445	−0,052	0,560
Albumin	0,100	0,068	0,071	0,195	0,090	0,100
CK	0,182	0,028	0,209	0,012	0,209	0,012
NA	−0,018	0,737	−0,021	0,693	−0,019	0,717
K	0,165	0,002	0,181	0,001	0,178	0,001
CA	0,052	0,329	0,036	0,502	0,045	0,392
CRP	−0,158	0,003	−0,137	0,010	−0,149	0,005
WBC	0,125	0,017	0,079	0,135	0,106	0,044
RBC	0,116	0,027	0,110	0,038	0,115	0,029
HG	0,154	0,003	0,159	0,002	0,157	0,003
HCT	0,132	0,012	0,144	0,006	0,139	0,008
PLT	0,001	0,996	−0,028	0,602	−0,010	0,850
Neutrophil	0,115	0,030	0,077	0,146	0,102	0,054
Lymphocyte	0,072	0,172	0,019	0,719	0,045	0,390
PT	−0,051	0,339	−0,036	0,502	−0,047	0,372
PTT	−0,074	0,164	−0,035	0,506	−0,058	0,271
INR	−0,019	0,714	0,001	0,981	−0,012	0,814

**Table 8:** Clinical parameters of survivors and dead patients at follow-up period in ICU.

	Recovery	Exitus	*p*
*Gender *Median ± SD (median)	60,1 ± 18,7 (62)	64,5 ± 16,4 (68)	0,070
*Gender n* (%)			
Male	182 (67,2)	56 (61,5)	0,328
Female	89 (32,8)	35 (38,5)	
*Cigarette*	170 (62,7)	57 (62,6)	1,000
*Additional diseases n* (%)			
Diabetes	80 (29,5)	36 (39,6)	0,076
Dyspnea	142 (52,4)	84 (92,3)	<0,001
Renal insufficiency	72 (26,6)	50 (54,9)	<0,001
Cancer	123 (45,4)	33 (36,3)	0,128
KKY	81 (29,9)	34 (37,4)	0,185
KOAH	40 (14,8)	28 (30,8)	0,001
*Transfusion n* (%)	155 (57,2)	65 (71,4)	0,016
*Operation n* (%)	202 (74,5)	31 (34,1)	<0,001
*Operation n* (%)			
Urgent	87 (32,1)	23 (25,3)	<0,001
Elective	115 (42,4)	8 (8,8)	
None	69 (25,5)	60 (65,9)	
*Type of Anesthesia n* (%)			
General	189 (69,7)	30 (33,0)	<0,001
Regional	13 (4,8)	1 (1,1)	
None	69 (25,5)	60 (65,9)	
*Clinic n* (%)			
Urgent	36 (13,3)	26 (28,6)	<0,001
Surgical	208 (76,8)	39 (42,9)	
Internal	27 (10,0)	26 (28,6)	
*ASA Score n* (%)	2,4 ± 0,9 (2)	2,9 ± 1,1 (3)	0,011
1	39 (18,9)	5 (13,2)	0,023
2	67 (32,5)	8 (21,1)	
3	77 (37,4)	13 (34,2)	
4	21 (10,2)	11 (28,9)	
5	2 (1,0)	1 (2,6)	
*GCS *mean ± SD (Min–Max)	13,9 ± 2,6 (15)	11,5 ± 4,1 (13)	<0,001
0–8	20 (7,4)	20 (22,2)	<0,001
>9	249 (92,6)	70 (77,8)	
*Ventilator need (day) *Ave. ± SD (Min–Max)	10,6 ± 35,0 (3)	18,4 ± 27,6 (7)	<0,001
*Duration of stay *Ave. ± SD (Min–Max)	7,2 ± 21,7 (2)	19,5 ± 29,2 (8)	<0,001

**Table 9:** Laboratory parameters of survivors and dead patients at follow-up period in ICU.

	Recovery	Exitus	
	Ave. ± SD (median)	Ave. ± SD (median)	*p*
Glucose	152,1 ± 58,5 (139)	152,7 ± 95,6 (128,5)	0,136
Urea	59,6 ± 61,0 (39)	85,0 ± 49,0 (77,5)	<0,001
Creatinine	1,61 ± 2,42 (0,95)	1,84 ± 1,53 (1,11)	0,003
AST	105,5 ± 337,6 (31)	189,6 ± 449,9 (40)	0,017
ALT	71,4 ± 232,3 (20)	93,9 ± 170,5 (27)	0,126
GGT	67,2 ± 104,8 (31)	94,7 ± 160,6 (50,5)	0,005
ALP	102,2 ± 109,2 (69)	118,2 ± 90,0 (80,6)	0,002
T. Protein	5,0 ± 1,0 (5)	5,0 ± 1,0 (5)	0,762
Albumin	2,6 ± 0,6 (2,6)	2,4 ± 0,6 (2,4)	0,012
CK	294,9 ± 570,6 (137)	172,6 ± 226,8 (83,5)	0,086
NA	136,0 ± 8,3 (137)	137,6 ± 7,3 (138)	0,118
K	4,4 ± 3,4 (4,1)	4,4 ± 1,0 (4,2)	0,170
CA	7,8 ± 0,9 (7,85)	7,7 ± 1,1 (7,7)	0,106
CRP	114,8 ± 106,4 (88,5)	140,9 ± 111,7 (111)	0,050
WBC	13,7 ± 8,5 (12,5)	17,8 ± 15,6 (12,64)	0,165
RBC	3,9 ± 0,8 (4)	3,6 ± 0,8 (3,55)	0,001
HG	10,7 ± 2,1 (10,9)	9,7 ± 2,3 (9,6)	<0,001
HCT	32,9 ± 6,4 (33,2)	30,3 ± 7,0 (30)	0,001
PLT	242239,9 ± 132297,6 (225000)	192876,4 ± 168797,7 (158000)	<0,001
Neutrophil	11,5 ± 7,4 (10,25)	15,3 ± 14,1 (10,9)	0,157
Lymphocyte	1,26 ± 1,21 (1)	1,42 ± 1,55 (1)	0,700

**Table 10:** Multivariate regression analysis of predictors for mortality.

Variables	Odds ratio (95% confidence interval)	*p*
COPD	0.307 (0.113–0.835)	0.021
Total PMA	0.812 (0.741–0.890)	<0.000

## References

[1] Le Maguet P., Roquilly A., Lasocki S. (2014). Prevalence and impact of frailty on mortality in elderly ICU patients: A prospective, multicenter, observational study. *Intensive Care Medicine*.

[2] Vincent J.-L., Moreno R. (2010). Clinical review: scoring systems in the critically ill. *Critical Care*.

[3] Ravn B., Prowle J. R., Mårtensson J., Martling C.-R., Bell M. (2017). Superiority of Serum Cystatin C Over Creatinine in Prediction of Long-Term Prognosis at Discharge From ICU. *Critical Care Medicine*.

[4] Docherty A. B., Sim M., Oliveira J. (2017). Early troponin I in critical illness and its association with hospital mortality: A cohort study. *Critical Care*.

[5] Zuckerman J., Ades M., Mullie L. (2017). Psoas Muscle Area and Length of Stay in Older Adults Undergoing Cardiac Operations. *The Annals of Thoracic Surgery*.

[6] Fairchild B., Webb T. P., Xiang Q., Tarima S., Brasel K. J. (2015). Sarcopenia and frailty in elderly trauma patients. *World Journal of Surgery*.

[7] Weijs P. J. M., Looijaard W. G. P. M., Dekker I. M. (2014). Low skeletal muscle area is a risk factor for mortality in mechanically ventilated critically ill patients. *Critical Care*.

[8] Moisey L. L., Mourtzakis M., Cotton B. A. (2013). Skeletal muscle predicts ventilator-free days, ICU-free days, and mortality in elderly ICU patients. *Critical Care*.

[9] Mueller N., Murthy S., Tainter C. R. (2016). Can sarcopenia quantified by ultrasound of the rectus femoris muscle predict adverse outcome of surgical intensive care unit patients as well as frailty? a prospective, observational cohort study. *Annals of Surgery*.

[10] von Haehling S., Anker M. S., Anker S. D. (2016). Prevalence and clinical impact of cachexia in chronic illness in Europe, USA, and Japan: facts and numbers update 2016. *Journal of Cachexia, Sarcopenia and Muscle*.

[11] Marra A. M., Arcopinto M., Bossone E., Ehlken N., Cittadini A., Grünig E. (2015). Pulmonary arterial hypertension-related myopathy: An overview of current data and future perspectives. *Nutrition, Metabolism & Cardiovascular Diseases*.

[12] Sheetz K. H., Zhao L., Holcombe S. A. (2013). Decreased core muscle size is associated with worse patient survival following esophagectomy for cancer. *Diseases of the Esophagus*.

[13] Finn M., Green P. (2015). The Influence of Frailty on Outcomes in Cardiovascular Disease. *Revista espanola de cardiologia (English ed.)*.

[14] Song X., Mitnitski A., Rockwood K. (2010). Prevalence and 10-Year outcomes of frailty in older adults in relation to deficit accumulation. *Journal of the American Geriatrics Society*.

[15] Rockwood K., Howlett S. E., MacKnight C. (2004). Prevalence, Attributes, and Outcomes of Fitness and Frailty in Community-Dwelling Older Adults: Report From the Canadian Study of Health and Aging. *The Journals of Gerontology. Series A, Biological Sciences and Medical Sciences*.

[16] Shamliyan T., Talley K. M. C., Ramakrishnan R., Kane R. L. (2013). Association of frailty with survival: A systematic literature review. *Ageing Research Reviews*.

[17] Fried L. P., Ferrucci L., Darer J., Williamson J. D., Anderson G. (2004). Untangling the Concepts of Disability, Frailty, and Comorbidity: Implications for Improved Targeting and Care. *The Journals of Gerontology. Series A, Biological Sciences and Medical Sciences*.

[18] Kim H., Hirano H., Edahiro A. (2016). Sarcopenia: Prevalence and associated factors based on different suggested definitions in community-dwelling older adults. *Geriatrics & Gerontology International*.

[19] Shah N., Abeysundara L., Dutta P. (2017). The association of abdominal muscle with outcomes after scheduled abdominal aortic aneurysm repair. *Anaesthesia*.

[20] Fielding R. A., Vellas B., Evans W. J. (2011). Sarcopenia: an undiagnosed condition in older adults. Current consensus definition: prevalence, etiology, and consequences. International working group on sarcopenia. *Journal of the American Medical Directors Association*.

[21] Heymsfield S. B., Adamek M., Gonzalez M. C., Jia G., Thomas D. M. (2014). Assessing skeletal muscle mass: Historical overview and state of the art. *Journal of Cachexia, Sarcopenia and Muscle*.

[22] Shen W., Punyanitya M., Wang Z. (2004). Total body skeletal muscle and adipose tissue volumes: estimation from a single abdominal cross-sectional image. *Journal of Applied Physiology*.

[23] Mourtzakis M., Prado C. M. M., Lieffers J. R., Reiman T., McCargar L. J., Baracos V. E. (2008). A practical and precise approach to quantification of body composition in cancer patients using computed tomography images acquired during routine care. *Applied Physiology, Nutrition, and Metabolism*.

[24] Gomez-Perez S. L., Haus J. M., Sheean P. (2016). Measuring abdominal circumference and skeletal muscle from a single cross-sectional computed tomography image: A step-by-step guide for clinicians using National Institutes of Health ImageJ. *Journal of Parenteral and Enteral Nutrition*.

[25] Mayberry R. M., Nicewander D. A., Qin H., Ballard D. J. (2017). Improving Quality and Reducing Inequities: A Challenge in Achieving Best Care. *Baylor University Medical Center Proceedings*.

[26] Bickenbach J., Fries M., Rex S. (2011). Outcome and mortality risk factors in long-term treated ICU patients: A retrospective analysis. *Minerva Anestesiologica*.

[27] Hopkins T. J., Raghunathan K., Barbeito A. (2016). Associations between ASA Physical Status and postoperative mortality at 48 h: a contemporary dataset analysis compared to a historical cohort. *Perioperative Medicine*.

[28] Wolters U., Wolf T., Stützer H., Shcr T., Shcröder T. (1996). Associations between ASA Physical Status and postoperative mortality at 48 h: a contemporary dataset analysis compared to a historical cohort. *British Journal of Anaesthesia*.

[29] Sankar A., Johnson S. R., Beattie W. S., Tait G., Wijeysundera D. N. (2014). Reliability of the American Society of Anesthesiologists physical status scale in clinical practice. *British Journal of Anaesthesia*.

[30] Sobol J. B., Wunsch H. (2011). Triage of high-risk surgical patients for intensive care. *Critical Care*.

[31] Uzman S., Yilmaz Y., Toptas M. (2016). A retrospective analysis of postoperative patients admitted to the intensive care unit. *Hippokratia*.

[32] Abbas S., Kahokher A., Mahmoud M., Hill A. (2008). Physiologic Modification of the American Society of Anaesthesiology Score (ASA) for Prediction of Morbidity and Mortality after Emergency Laparotomy. *The Internet Journal of Surgery*.

[33] Zali A. R., Seddighi A. S., Seddighi A., Ashrafi F. (2012). Comparison of the acute physiology and chronic health evaluation score (APACHE) II with GCS in predicting hospital mortality of neurosurgical intensive care unit patients.. *Global Journal of Health Science*.

[34] Harrison D. A., Lone N. I., Haddow C. (2014). External validation of the Intensive Care National Audit Research Centre (ICNARC) risk prediction model incritical care units in Scotland. *BMC Anesthesiol*.

[35] Minto G., Biccard B. (2014). Assessment of the high-risk perioperative patient. *Continuing Education in Anaesthesia, Critical Care and Pain*.

[36] Pearse R. M., Moreno R. P., Bauer P. (2012). European Surgical Outcomes Study (EuSOS) group for the Trials groups of the European Society of Intensive Care Medicine and the European Society of Anesthesiology. *Mortality after surgery in Europe: a 7 day cohort study*.

[37] Higgins T. L. (2007). Quantifying risk and benchmarking performance in the adult intensive care unit. *Journal of Intensive Care Medicine*.

[38] Kalyani R. R., Corriere M., Ferrucci L. (2014). Age-related and disease-related muscle loss: the effect of diabetes, obesity, and other diseases. *The Lancet Diabetes & Endocrinology*.

[39] Fan J., Kou X., Yang Y., Chen N. (2016). MicroRNA-regulated proinflammatory cytokines in sarcopenia. *Mediators of Inflammation*.

[40] Lutz C. T., Quinn L. S. (2012). Sarcopenia, obesity, and natural killer cell immune senescence in aging: altered cytokine levels as a common mechanism. *AGING*.

[41] Umegaki H. (2015). Sarcopenia and diabetes: Hyperglycemia is a risk factor for age-associated muscle mass and functional reduction. *Journal of Diabetes Investigation*.

[42] Morley J. E., Malmstrom T. K., Rodriguez M. (2014). Frailty is also higher in the prevalence in older with DM. *Journal of the American Medical Directors Association*.

[43] Kalyani R. R., Metter E. J., Egan J., Golden S. H., Ferrucci L. (2015). Hyperglycemia predicts persistently lower muscle strength with aging. *Diabetes Care*.

